# The impact of lockdown on Aragonese children: a geographical analysis of childhood obesity pre- and post-lockdown

**DOI:** 10.3389/fpubh.2025.1696722

**Published:** 2026-01-16

**Authors:** Alba Gállego-Royo, Carmen Bentué-Martinez, María Zúñiga Antón, Ana Gastón-Faci, Nuria Val-Jimenez, Bárbara Marco-Gomez, Rosa Magallón-Botaya

**Affiliations:** 1 Preventive Medicine Resident, Miguel Servet University Hospital, Aragonese Health Service, Zaragoza, Spain; 2 H36_23D Feminization and Ethics of the Health Professions Group (FEPS), IIS Aragón, Zaragoza, Spain; 3 GIS011 Primary Care Research Group, IIS Aragón, Zaragoza, Spain; 4 GIS076 Spatial Planning Studies Group, University Institute of Environmental Sciences of Aragon (IUCA), University of Zaragoza, IIS Aragon, Zaragoza, Spain; 5 Utebo Health Centre, Aragonese Health Service, Zaragoza, Spain; 6 B21_23R Aragonese Primary Care Research Group (GAIAP), Zaragoza, Spain; 7 Family Medicine, Actur Sur Health Centre, Aragonese Health Service, Zaragoza, Spain; 8 Royo Villanova University Hospital, Aragonese Health Service, Zaragoza, Spain; 9 Department of Medicine, Psychiatry and Dermatology, Faculty of Medicine, University of Zaragoza, Zaragoza, Spain

**Keywords:** child, geographic mapping, obesity, paediatric obesity, socioeconomic factors

## Abstract

**Background:**

Childhood obesity remains a major public health concern, exacerbated by the COVID-19 pandemic. This study examines temporal and spatial patterns in childhood obesity and overweight in Aragón, Spain, before (2018–2019) and after (2021) pandemic lockdowns.

**Methods:**

We conducted a retrospective descriptive analysis using anthropometric data from children aged 0–14 years attending public health centres. Data were extracted from electronic medical records and classified according to national diagnostic standards. Sociodemographic indicators, including parental education and income, were derived from official datasets and geoprocessed to the Basic Health Zone (BHZ) level. Principal Component Analysis (PCA) reduced socioeconomic dimensions, whilst Geographically Weighted Regression (GWR) examined spatial associations.

**Results:**

Obesity prevalence increased from 3.8% in 2019 to 5.5% in 2021, and overweight from 14.5 to 15.8% (*p* < 0.001). Boys were consistently more affected than girls, though this gap narrowed post-pandemic. The largest increases were amongst children aged >6 years. Urban–rural typology alone did not fully explain the patterns; instead, intermediate-population BHZs and rural areas near urban centres exhibited higher prevalence. GWR analyses revealed spatial variability in the relationship between obesity and socioeconomic indicators, particularly in Zaragoza province, with income and education levels significantly associated with prevalence.

**Conclusion:**

The COVID-19 pandemic reversed prior improvements in childhood obesity trends in Aragón. Socioeconomic inequalities, especially related to parental education and income, strongly influence obesity distribution at the local level. Geographically targeted policies are needed to reduce disparities and prevent long-term health consequences in children.

## 1 Introduction

Childhood obesity is a severe public health issue that must be urgently addressed. It represents a risk factor for non-communicable diseases in adulthood, highlighting the importance of surveillance and institutional commitment in implementing measures for its control (1). Current figures from the World Health Organization (WHO) are alarming: 29.5% of children aged 5–9 years are overweight or obese, as well as 24.9% of those aged 10–19 years (2). Mediterranean regions report the highest prevalence rates.

Within the European context, the WHO has been monitoring childhood obesity since 2007 (3). This initiative collects data from 45 European countries, including Spain since 2009. Alarmingly, the southern regions (Cyprus, Greece, Italy, and Spain) exhibit the highest rates, with obesity prevalence in boys ranging from 18 to 24% and overweight from 20 to 24%. Amongst girls, obesity rates range from 13 to 14%, and overweight prevalence from 23 to 26%. In contrast, Northern European countries such as Denmark report an obesity prevalence of 6% (4).

Focusing on Spain, the WHO estimates the combined prevalence of obesity and overweight amongst children aged 5–19 years to be between 31.9 and 37.9% (2). The 2020 National Health Survey, which includes children aged 2–17 years, reports an obesity rate of 28.56% (5). In Aragón, prevalence is lower, at 20.71%. A rising trend was observed until 2010, with overweight at 9.2% and obesity at 10.4% in a study using 2011 data for children aged 2–14 years (6). Subsequently, a slight decline was observed until 2015 (7), coinciding with national and international community-based interventions promoting healthy eating and physical activity. These initiatives, involving families and schools, have shown measurable improvements in children’s lifestyle behaviours and modest reductions in overweight and obesity prevalence.

Since 2020, the COVID-19 pandemic and its associated measures—such as the national lockdown in Spain from 14 March to 3 June 2020 and school closures—seem to have reversed this downward trend, potentially returning to levels seen decades ago (8, 9). Whilst these measures impacted all population groups, children, particularly those in vulnerable settings (defined as areas with social or environmental characteristics that increase health risks and limit access to protective resources), may have been disproportionately affected. Schools are protective factors in vulnerable areas, supporting the maintenance of healthy lifestyles. Studies highlight that school holidays, during which school meals and physical activities are unavailable, are associated with an increase in BMI amongst children (8, 10).

Analysing childhood obesity, its prevention, and treatment requires a biopsychosocial approach. Adherence to healthy dietary habits is influenced by various factors, particularly the family’s socioeconomic status (11, 12). Living in areas with low socioeconomic levels is a predictor of poor adherence (13). Additionally, mental health conditions such as depression have been identified as risk factors, especially regarding childhood obesity and its management (14).

Childhood obesity is a multifactorial problem with numerous risk factors (15), exhibiting significant territorial differences in legislation and regulatory frameworks. Aragón has particular characteristics: an autonomous community with over 1.3 million inhabitants, accounting for nearly 3% of the Spanish population (16). Over 50% of Aragón’s population resides in Zaragoza and its metropolitan area, with the remaining population distributed across numerous sparsely populated settlements. It is a Spanish region with similarities to some northern European areas, such as northern Finland, Sweden, or Norway (17), as well as regions like Lika-Senj County in Croatia or Euritania in Greece (18), characterised by centralised hubs and extensive rural areas with low population density.

Health information systems in Aragón do not track the prevalence of childhood obesity globally or within specific health zones. However, all children aged 0–14 years are routinely assessed through the regional Healthy Child Programme (RNS) (19), which forms part of the Primary Care Services Portfolio. During these scheduled visits, paediatricians systematically record weight, height, and other developmental indicators, ensuring standardised and comparable anthropometric measurements across the region. Detailed analysis of childhood obesity and overweight based on the child’s place of residence therefore allows the identification of area-level characteristics [e.g., socioeconomic, relational, and locational features of each Basic Health Zone (BHZ) (20)] that may contribute to this prevalence.

To the best of our knowledge, this geographically referenced influence, along with the impact of extraordinary events such as the COVID-19 pandemic (21), has not been studied.

Our objective is to determine the prevalence of obesity and overweight in children aged 0–14 years, based on RNS visits conducted in primary care health centres in Aragon during the pre-pandemic period (between January 2018 and December 2019) and post-lockdown (between January and December 2021). These visits form part of the standard preventive care schedule of the public health system. Weight and height measurements taken during these periods will be analysed, considering both sex and age. Additionally, we aim to identify differences in obesity and overweight prevalence across various sociodemographic areas, with particular attention to disparities between rural and urban zones.

## 2 Methods

A retrospective descriptive study was conducted to examine the prevalence of obesity and overweight at two time points (2018–2019 and 2021) using data extracted from primary care medical records within the national health system. Variables analysed included weight, height, age, sex, BHZ (the spatial unit of analysis in this study) and the three provinces of Aragón (Huesca, Zaragoza, and Teruel). BHZs are the geographical units used by the Spanish National Health System to divide the territory for the distribution and organization of healthcare services (16).

Based on the collected data, patients were classified as obese, overweight, or having a normal weight according to diagnostic criteria for the Spanish population [Faustino Orbegozo Eizaguirre Foundation criteria, 2011 (22)]. Age categories were established based on educational stages: 0–3 years (pre-school), 3–6 years (early childhood), 6–12 years (primary), and 12–14 years (secondary school). The territorial typology indicator is classified into three categories based on population size: urban BHZs (10,000 inhabitants or more), intermediate BHZs (2,000–10,000 inhabitants), and rural BHZs (fewer than 2,000 inhabitants). Urban BHZs correspond to provincial capitals (the cities of Huesca, Zaragoza, and Teruel), the metropolitan area surrounding Zaragoza, and corridors to the east and west of the autonomous community. Intermediate BHZs cover much of the remaining area in the provinces of Zaragoza, Huesca, and the northeastern part of Teruel. Lastly, rural BHZs are predominantly located in the province of Teruel and the northern region of Huesca.

Data was anonymised. Social determinants (SD) analysed were sourced from the Household Income Distribution Atlas (net median income per person for 2020) and the 2011 Population and Housing Census (education level indicators), provided by the Spanish National Statistics Institute [INE (23)]. Originally disaggregated at the census tract level, these indicators were aggregated to the BHZ level through vector-based geoprocessing operations.

The dataset used in this study does not represent a sample but the complete set of records from all children aged 0–14 years in Aragón who attended the RNS in primary care health centres during the study periods. Therefore, no sample size calculation was required. The data extraction provided absolute values for the entire autonomous community, covering all BHZs. Table 1 presents the total number of records obtained, including the sex distribution, together with the age distribution of the population according to the INE (23).

**Table 1:** Population of children aged 0–14 years in Aragón and number of recorded measurements in 2018–2019 and 2021.

Population	Population of Aragón as of 1/07/2021		Number of children with recorded measurements obtained 2021		Population of Aragón as of 1/01/2019		Number of children with recorded measurements obtained 2018–2019	
	Male	Female	Male	Female	Male	Female	Male	Female
Population of children aged 0–14 years	92,968	87,251	38,098	37,154	96,110	90,333	58,624	56,427
	180,220		75,252		186,443		115,051	

Population of Aragón aged 0–14 years and number of children with recorded anthropometric measurements in 2018–2019 and 2021, stratified by sex according to data from the National Institute of Statistics (INE) (23).

### 2.1 Statistical analysis

Statistical analysis was performed using SPSS 25.0. Initial data cleaning excluded cases with incongruent weight, height, or BMI values for participants’ ages. This was necessary as the data were manually entered by paediatric and family medicine professionals conducting the assessments, leading to occasional errors in unit transcription (e.g., grammes instead of kilogramems) or misrecording of height as weight and vice versa. All analyses were conducted separately for each study period.

A descriptive analysis of quantitative variables was performed using median and interquartile range, whilst qualitative variables were summarised as absolute values and percentages. Prevalence rates were calculated globally, followed by bivariate analysis of diagnosis (obesity/overweight) and variables such as sex, age categories, and rural/urban classification. Pearson’s *χ*^2^ test was used for dichotomous qualitative variables, and Student’s t-test for quantitative variables. For qualitative variables with more than two categories, χ^2^ tests with Bonferroni correction were applied for pairwise comparisons.

### 2.2 Multivariate analysis

A Principal Component Analysis (PCA) was conducted to reduce the dimensionality of the original data and address collinearity amongst SD indicators. PCA identifies linear combinations of indicators that capture most of the original information, representing it in new axes (principal components). The first principal component accounts for the greatest variance, the second for the remaining variance, and so on, up to the number of original variables. Components were selected using Kaiser’s criterion (components with eigenvalues >1) (24).

Spatial variability in the association between prevalence and SDs was analysed using Geographically Weighted Regression (GWR) (25). GWR defines a region around each spatial entity 𝑖 (with coordinates 𝑢𝑖, 𝑣𝑖), using data points within a specified neighbourhood to calibrate a regression model for that location. This process generates local regression statistics for each location. GWR applies a distance-based weighting model, giving greater weight to observations nearer the centre of the window. The following equation describes a conventional GWR model:


yi=∑kβk(ui,vi)xk.i+εi

Where yi, xk,i, and εi are, respectively, a dependent variable, the k-th independent variable, and the Gaussian error at location i; (ui, vi) is the x-y coordinate of the i-th location; and the coefficients *β* (ui, vi) vary across space. GWR4.09 software was used for this analysis.

The project complies with Law 14/2007 and the Declaration of Helsinki and has been approved by the Aragón Research Ethics Committee (PI22/240).

## 3 Results

The prevalence of childhood obesity in Aragón was 3.8% in 2019, rising to 5.5% in 2021. Similarly, overweight prevalence increased from 14.5% in 2019 to 15.8% in 2021, with differences statistically significant (*p* < 0.001). In addition, using normal weight as the reference category, the odds of childhood obesity in 2021 were significantly higher than in 2019 (OR = 1.50), and the odds of overweight also showed a moderate increase (OR = 1.13).

Statistically significant differences (*p* < 0.001) were found in both periods for sex and the likelihood of being overweight or obese (Table 2). Boys were more likely to be overweight or obese than girls in both 2019 (*p* < 0.001) and 2021 (*p* < 0.001). Following the COVID-19 pandemic, obesity rates increased in both sexes, with girls’ prevalence matching that of boys, a change from prior years.

**Table 2:** Comparative analysis of diagnosis frequencies by BMI between 2018–2019 and 2021.

2018–2019										
Weight status		Gender		Age				Type of entity		
		Male	Female	0–3	3–6	6–12	>12	Rural	Intermediate	Urban
Normal weight	*N*	46,137	47,894	18,223	29,624	36,423	9,761	1,161	12,572	80,298
	% (CI 95%)	78.7% (78.4–79)	84.9% (84.6–85.2)	90.7% (90.3–91.1)	84.7% (84.3–85.1)	77.7% (77.3–78.1)	74.5% (73.8–75.3)	82.7% (80.6-84.6)	81.5% (80.9-82.1)	81.8% (81.5–82.0)
Overweight	*N*	9,931	6,723	1,587	4,209	8,302	2,556	188	2,238	14,228
	% (CI 95%)	16.9% (16.6–17.2)	11.9% (11.6–12.2)	7.9% (7.5–8.3)	12.0% (11.7–12.4)	17.7% (17.4–18.1)	19.5% (18.8–20.2)	13.4% (11.7–15.2)	14.5% (14.0–15.1)	14.5% (14.3–14.7)
Obesity	*N*	2,556	1,810	284	1,139	2,165	778	55	618	3,693
	% (CI 95%)	4.4% (4.2–4.5)	3.2% (3.2–3.4)	1.4% (1.3–1.6)	3.3% (3.1–3.4)	4.6% (4.4–4.8)	5.9% (5.5–6.4)	3.9% (3.0–5.0)	4.0% (3.7–4.3)	3.8% (3.6–3.9)

2021										
Weight status		Gender		Age				Type of entity		
		Male	Female	0–3	3–6	6–12	>12	Rural	Intermediate	Urban
Normal weight	*N*	28,791	30,453	14,546	16,077	19,824	8,797	839	7,676	50,729
	% (CI 95%)	75.6% (75.1–76)	82.0% (81.6–82.4)	89.3% (88.9–89.8)	80.7% (80.1–81.2)	73.0% (72.4–73.5)	74.1% (73.3–74.8)	78.6% (76.0–80.9)	78.4% (77.6–79.2)	78.8% (78.5–79.1)
Overweight	*N*	6,901	4,982	1,415	2,720	5,541	2,207	172	1,562	10,149
	% (CI 95%)	18.1% (17.7–18.5)	13.4% (13.1–13.8)	8.7% (8.3–9.1)	13.6% (13.2–14.1)	20.4% (19.9–20.9)	18.6% (17.9–19.3)	16.1% (14.0–18.4)	15.9% (15.2–16.7)	15.8% (15.5–16.0)
Obesity	*N*	2,406	1,719	321	1,131	1,799	874	57	556	3,512
	% (CI 95%)	6.3% (6.1–6.6)	4.6% (4.4–4.8)	2.0% (1.8–2.2)	5.7% (5.4–6)	6.6% (6.3–6.9)	7.4% (6.9–7.8)	5.3% (4.1–6.8)	5.7% (5.2–6.1)	5.5% (5.3–5.6)

Percentage distribution and 95% confidence intervals of normal weight, overweight and obesity in 2018–2019 and 2021, stratified by sex, age group and type of entity (rural, intermediate, urban). Classifications were based on the Orbegozo 2011 growth standards.

**Figure 1:**
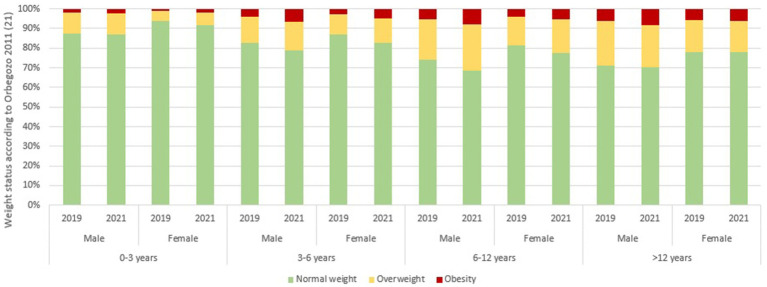
Weight status distribution by age group and sex in 2019 and 2021 according to Orbegozo 2011 criteria. Stacked bar charts showing the percentage of children classified as normal weight, overweight and obese in 2019 and 2021, stratified by sex and age group (0–3, 3–6, 6–12, and >12 years). Weight status categories were defined according to the Orbegozo 2011 growth references (21).

Analysing educational age groups revealed that in 2019, obesity was most prevalent amongst pre-adolescents (aged >12 years) at 5.9%, followed by primary-aged children (6–12 years) at 4.6%. These differences were statistically significant (*p* < 0.001). By 2021, obesity rates increased across all age groups, with the highest rates amongst children aged 6–12 years (6.6%) and those aged >12 years (7.4%). Comparing the combined prevalence of overweight and obesity amongst boys and girls, a progressive increase was observed between 2019 and 2021 in children aged 6–12 years and >12 years, with statistically significant differences (*p* < 0.001). The pre-school age group (0–3 years) was the only category without statistically significant differences (Figure 1).

The temporal trend in overweight and obesity percentages increased between 2019 and 2021. However, focusing on residence types, intermediate zones showed the highest figures, though differences were not statistically significant (2019 *p* = 0.461; 2021 *p* = 0.870). Age and sex were not included in the regression, as BMI prevalence for each BHZ was adjusted for these variables using percentile-based comparisons.

### 3.1 Geographical analysis

Geographical Pattern Derived from the Analysis.

#### 3.1.1 Description of the figure depicting study area characterisation and prevalence based on PCA results

**Figure 2:**
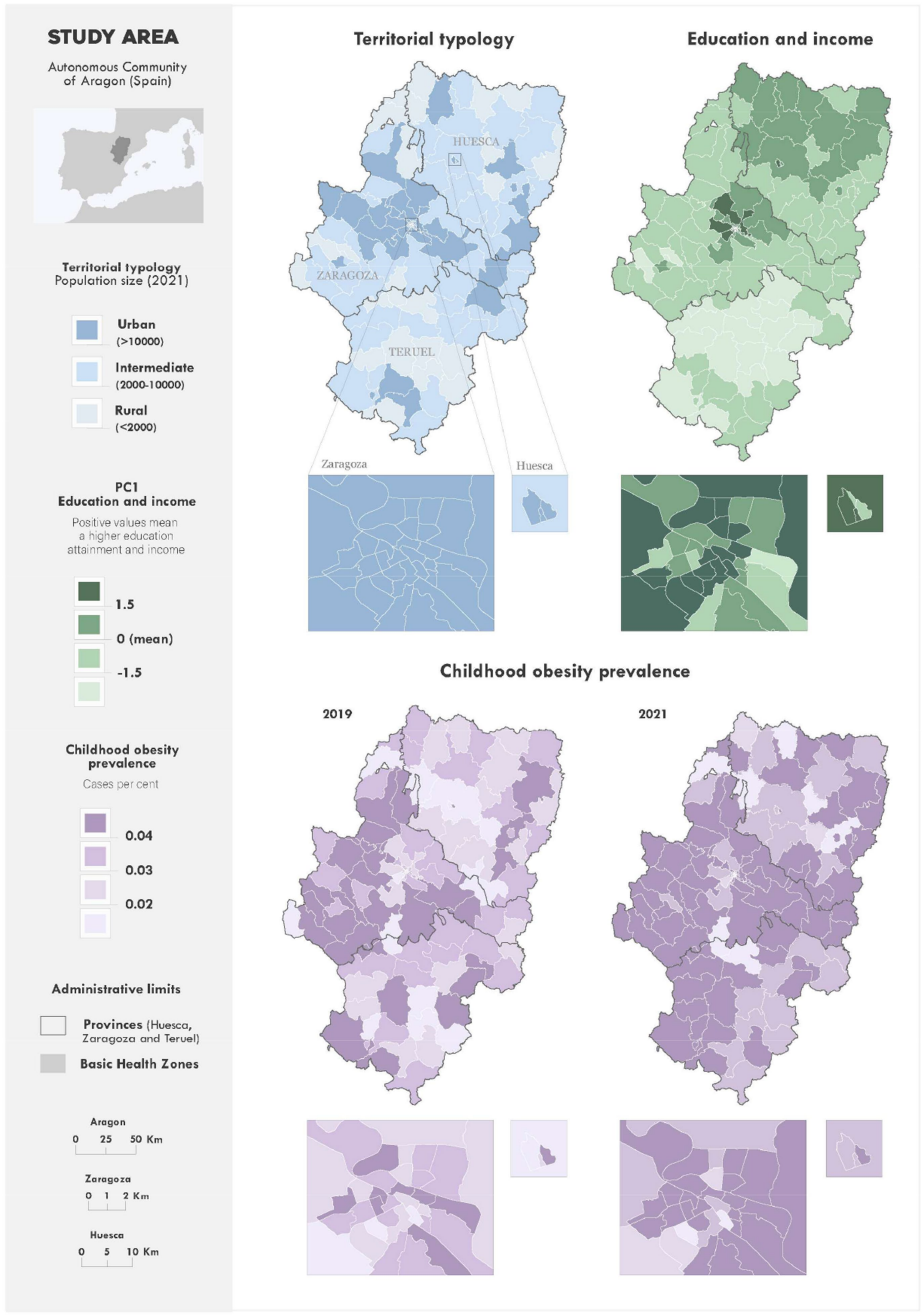
Spatial distribution of childhood obesity and socioeconomic indicators in Aragón: PCA and territorial typology analysis. Socioeconomic indicator (PC1), obesity prevalence, and territorial typology across BHZs. Social determinants used in the indicator were obtained from income and education data (INE) and aggregated from census tracts to BHZs.

Figure 2 characterises the study area through a cartographic representation of the indicators “territorial typology” and “education and income.” The latter is the first principal component derived from the PCA, serving as the explanatory variable for childhood obesity prevalence in the GWR regression model. Additionally, the spatial distribution of childhood obesity prevalence in 2019 and 2021 is shown. The administrative units depicted on the maps correspond to the BHZs.

The “education and income” indicator, an explanatory factor in the GWR regression model, represents the first principal component derived from PCA. It was selected based on Káiser’s criterion, showing a standard deviation greater than 1 (1.510) and accounting for 76% of cumulative variance. As this indicator results from linear combinations of original variables, it presents a range of positive and negative values, reflecting higher or lower levels of education and income, respectively. Higher levels of income and education are found in Huesca province and Zaragoza city and its metropolitan area, though notable internal variability is evident within Huesca and Zaragoza. Lower levels of income and education are concentrated in Teruel province.

Childhood obesity prevalence indicators share a common legend for both years. In 2019, the Zaragoza province—excluding BHZs in the metropolitan area of the city—exhibited the highest prevalence values. In Huesca and Teruel provinces, lower prevalence rates were observed on average, though some BHZs fell within the highest range. In the cities of Zaragoza and Huesca, average values were low overall, but corridors with elevated prevalence were evident. By 2021, there was a generalised increase in prevalence across the study area. Zaragoza province showed the largest number of BHZs with the highest prevalence values, joined by BHZs in the southwest and northeast of the region.

#### 3.1.2 Spatial variability in statistical association

**Figure 3:**
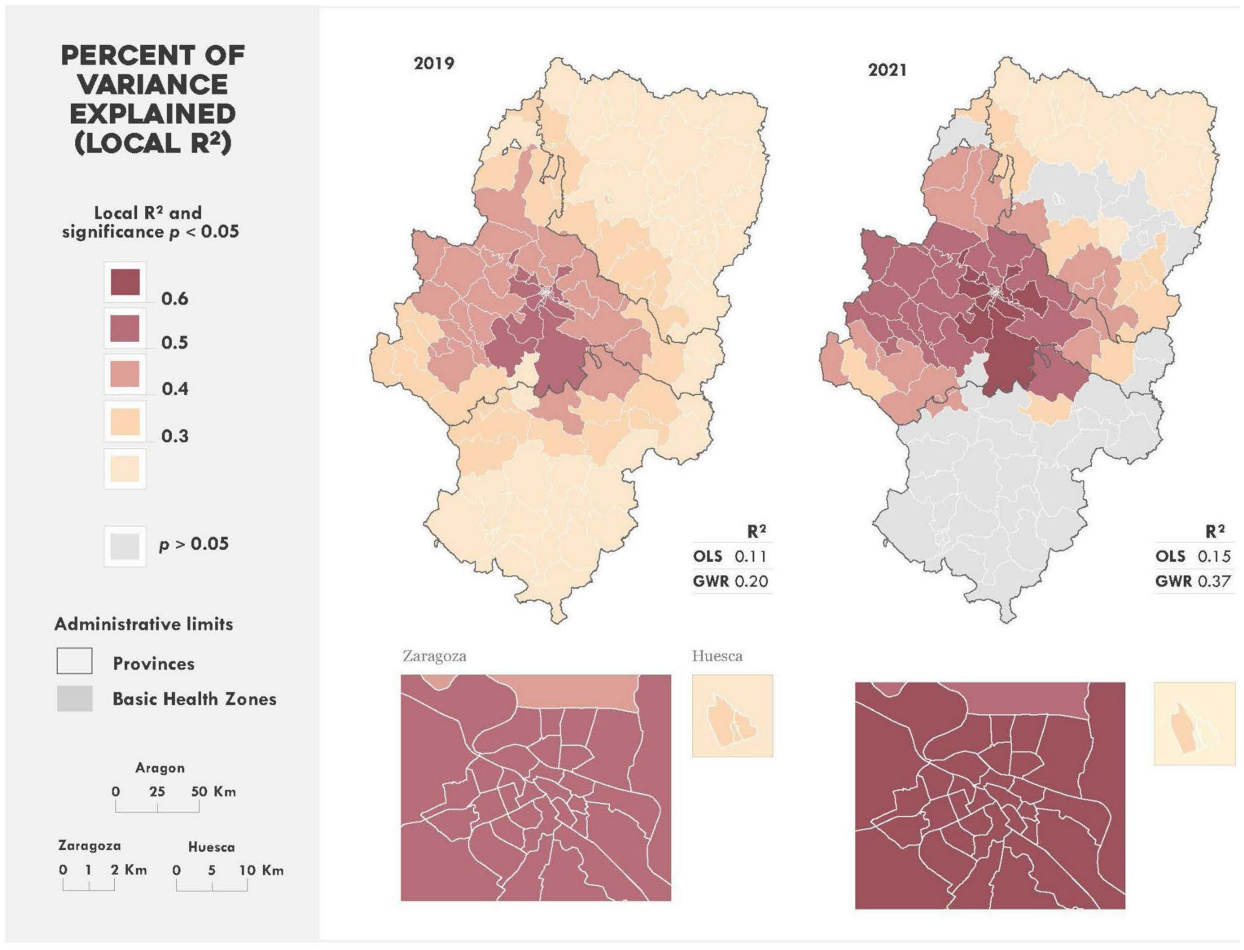
Spatial distribution of local *R*² values. Models for 2019 and 2021.

Regression analysis results indicate that the local GWR model outperformed the global Ordinary Least Squares (OLS) alternative in terms of *R*^2^ (Figure 3). The explanatory capacity of the “education and income” variable was higher for the 2021 prevalence model. The spatial distribution of local *R*^2^ was similar across both years, with BHZs in Zaragoza city, its metropolitan area, and the rest of the province showing the highest *R*^2^ values (ranging from 0.4 to 0.6). Most BHZs in Huesca and Teruel had lower local *R*^2^ values (<0.4) in both years. However, in 2019, all BHZs demonstrated significant values (*p* < 0.05), whilst in 2021, the *R*^2^ values for BHZs in Teruel and parts of Huesca were not significant (*p* > 0.05).

## 4 Discussion

Our study highlights a shift in trends compared to previous data, with an increase in childhood obesity and overweight prevalence following the pandemic. Notably, the data analysed were extracted from the RNS, conducted at primary care centres within the National Health System. These assessments are initiated by healthcare professionals for all patients attending consultations.

As shown in the data, there was a decline in childhood obesity trends before the pandemic, consistent with findings from García-Solano (7) and reports from the Gasol Foundation (PASOS Study 2019) (26), the DKV Foundation (27), and the National Health Survey (28). WHO, European, and Ministry of Health initiatives aimed at promoting physical activity, improving diets, and other measures contributed to this improvement. However, as Ruopeng warned (29), the pandemic reversed these trends, which is supported by national, European, and global data (9, 30), as well as our study’s findings. Recent national data from the ALADINO 2023 study (31) also suggest a potential downward trend in childhood overweight and obesity in the more recent post-pandemic period, although longer-term monitoring will be needed to confirm whether this pattern is sustained.

Children aged over 6 years showed a significant increase in obesity, likely tied to greater autonomy in food choices, reduced physical activity, and increased screen time (a trend exacerbated during the pandemic) (32). This pattern aligns with national and international findings. Non-school periods also posed nutritional risks for children in certain socioeconomic groups (8, 33, 34). Whilst this study does not address adolescents over 14 years old, studies by Moreno et al. suggest that obesity and overweight rates also increased in this group (35). Furthermore, other research evaluating a wider range of eating disorders during the pandemic highlighted that lockdown represented a particularly high and unique risk factor for adolescents (36, 37). Geographical analysis is essential to understand the influence of social and geographical factors on childhood obesity (38). Socioeconomic factors, particularly parental education and income levels, emerged as critical components (39, 40). International evidence has consistently shown that children living in households with lower educational attainment and income are more likely to present overweight and obesity(40). Lower socioeconomic BHZs showed higher obesity prevalence, and local GWR regression analysis clarified the spatial variability of these associations, especially post-pandemic in Zaragoza province. Previous studies have employed similar techniques to highlight the importance of addressing obesity at scales relevant to decision-making, ensuring that these scales are spatially adapted to the level of analysis required, whether more general or more detailed (41–44).

The geographic analysis presented in this study not only corroborates the patterns suggested by the simple rural–intermediate–urban classification but also reveals additional area-level vulnerabilities that are not captured by this categorisation alone. As shown in Figures 2, 3, several rural BHZs located near Zaragoza’s metropolitan area displayed a higher prevalence of childhood obesity compared with similarly populated zones at greater distance from urban centres, suggesting the influence of lifestyle, socioeconomic and environmental factors. This spatial perspective therefore highlights specific areas where children may be at higher risk and where targeted community-based or public health interventions could be prioritised. Furthermore, applying similar spatial approaches in other regions may allow policymakers to identify risk clusters, adapt interventions to local contexts, and design preventive programmes capable of mitigating the impact of future pandemics or disruptive events.

### 4.1 Limitations

Whilst participation in the RNS programme is high, it does not cover the entire 0–14 population in Aragón, excluding individuals in specific mutual insurance systems or those whose families opt out. Additionally, data entry errors from manual transcription necessitated a data-cleaning process to exclude outliers and incongruent entries. Nonetheless, official open-source data from the National Statistics Institute were used to derive socioeconomic indicators, ensuring reliability.

## 5 Conclusion

Childhood obesity prevalence has significantly increased following the SARS-CoV-2 pandemic. Socioeconomic determinants, particularly parental education and income, are crucial for understanding at-risk populations. These social factors elucidate differences at the BHZ level but are less evident at provincial or regional levels.

## Data Availability

The data analyzed in this study is subject to the following licenses/restrictions: Data were obtained from official sources of the Government of Aragon and cannot be publicly distributed. Requests to access these datasets should be directed to agallegoro@salud.aragon.es.
